# Comparison of Several Methods for Determining the Internal Resistance of Lithium Ion Cells

**DOI:** 10.3390/s100605604

**Published:** 2010-06-03

**Authors:** Hans-Georg Schweiger, Ossama Obeidi, Oliver Komesker, André Raschke, Michael Schiemann, Christian Zehner, Markus Gehnen, Michael Keller, Peter Birke

**Affiliations:** 1 Continental AG, Business Unit Hybrid and Electric Vehicles, Sickingenstrasse 42-46, 10553 Berlin, Germany; E-Mails: ossama.obeidi@continental-corporation.com (O.O.); michael.schiemann@continental-corporation.com (M.S.); michael.keller@continental-corporation.com (M.K.); peter.birke@continental-corporation.com (P.B.); 2 University of Applied Sciences Brandenburg, Magdeburger Strasse 50, 14770 Brandenburg, Germany; E-Mails: o.komesker@gmx.de (O.K.); zehner@fh-brandenburg.de (C.Z.); 3 Georg Agricola University of Applied Sciences, Herner Strasse 45, 44787 Bochum, Germany; E-Mails: raschke.andre@googlemail.com (A.R.); gehnen@tfh-bochum.de (M.G.)

**Keywords:** internal resistance, impedance, lithium-ion-cell, AC resistance, DC resistance

## Abstract

The internal resistance is the key parameter for determining power, energy efficiency and lost heat of a lithium ion cell. Precise knowledge of this value is vital for designing battery systems for automotive applications. Internal resistance of a cell was determined by current step methods, AC (alternating current) methods, electrochemical impedance spectroscopy and thermal loss methods. The outcomes of these measurements have been compared with each other. If charge or discharge of the cell is limited, current step methods provide the same results as energy loss methods.

## 1. Introduction

Capacity, internal resistance and self discharge are three main basic parameters determining the performance of lithium ion batteries in automotive applications. For a given battery voltage and weight, the specific energy of a battery is determined by its capacity, while the internal resistance limits its specific power. Heat generation in batteries is mainly caused by exothermic overpotential resistance, endothermic or exothermic heat by entropy change and exothermic heat caused by side reactions [1,2].

Under high current loads the heat evolution of the battery and energy efficiency are also primarily determined by its internal resistance. Therefore the precise knowledge of the internal resistance of a lithium ion battery is one of the most important factors for the design to specific applications.

Resistance is generally measured by applying a voltage to the device under test and measuring the resulting current or by applying a current and measuring the resulting voltage. Interpretation of the measurement result is easy, provided that the resistor or device under test is governed by Ohm’s law.

The definition of “internal resistance” for non-LTI (linear time invariant) systems is complex and unequivocal. While methods based on charge/discharge define “large signal resistance”, methods like impedance spectroscopy define “small signal resistance”. These two definitions are different for batteries (like other non-LTI systems) and could be connected with each other using system theory for non-linear system.

However, determining the internal resistance of complex loads is much more difficult because the outcome of the measurement is not only governed by the ohmic behavior of the device but also by its capacitive and inductive behavior. Things become even more complicated if additional non linearity such as temperature dependence and/or other time variant behavior of the device under test is provoked by the specific measurement procedure. Thus sophisticated measurement procedures have to be used for measuring the resistive part of a complex system. These procedures are based on the frequency dependency of the device under test. The measurement frequency is selected in such way that this frequency is high enough for the capacitive behavior of the device under test to not influence the outcome of measurement but at the same time is low enough that the inductance of the device under test does not influence it too much either. The measurement setup has to be designed carefully to avoid false results caused by the setup, e.g., parasitic capacity of cables may introduce a short cut at high frequencies.

A battery is a typical example of a complex load, showing capacitive, ohmic and inductive behavior. Additionally, its behavior is also strongly dependent on the measurement frequency and amplitude. Therefore, it is not easy to measure the internal resistance of a single cell or battery, which are representative examples of a nonlinear and time dependent system. Because of this behavior, the internal resistance of the cell is also a function of the method of determination [3]. Additionally, experimental conditions have an impact on the outcome of measurements too [3].

But, as a premise for successful battery application, the knowledge of the battery’s internal resistance is essential because this parameter is needed for dimensioning the battery system, for selecting and comparing cells, for energy efficiency calculation, for dimensioning the cooling system of the battery and for power estimation [4]. A proper design of the cooling system is necessary for safety and lifetime of a battery system [5]. It was therefore the goal of the present investigations to find a suitable method for measuring the internal resistance of batteries to cover these targets.

## 2. Theoretical Background

The cell voltage U_cell_ under load is governed by the open circuit voltage, the overvoltages caused by concentration polarisation η_diff_ and charge transfer polarisation η_ch,tr_ as well as the voltage drop caused by internal ohmic resistance R_i_ [3,6]:

(1) $$U_{\mathrm{cell}}=U_{\mathrm{ocv}}-\eta_{\mathrm{diff}}-\eta_{\mathrm{ch},tr}-{\mathrm{IR}}_{i}$$

The internal resistance of a battery is often quoted as a characteristic parameter. The meaning of the term “internal resistance” has to be considered with some caution because it is not a simple ohmic resistance and depends on the method used for its determination, on the state of charge of the battery and on the battery temperature [3]. In addition the “concentration polarisation” and “charge transfer polarisation” effects must be separated for voltage depression caused by ohmic internal resistance.

The ohmic resistance also encompasses the resistivity of the components of the battery such as the active material of the anode and the cathode, the current collectors and the electrolyte [7]. The internal resistance is further influenced by material contacts (e.g., between active material and the current collector), geometrical electrode (thickness, dimensions) and internal construction aspects. The effect of this part of the total effective resistance occurs very quickly, that means it can be seen during the first few milliseconds after a battery is placed under discharge or recharge [8] (see Figure 3).

Using “Ohm’s Law”, the total effective resistance is subsequently calculated by dividing the change in voltage by the change in current. This common method is described in literature [3,9,10]. The internal resistance is in series with the voltage of the battery, causing an internal voltage (change) drop. With no current flow, the voltage drop at the internal resistance is zero, thus, the voltage at the output terminals is governed by open circuit voltage. If a load is applied to the battery (positive or negative during charge and discharge), the load resistance is in series with internal resistance (R_i_) of the cell.

The magnitude of R_i_ is mainly determined by the processes at the interface between active material and electrolyte, the electrolyte conductivity and the loss by the purely ohmic content of the supporting and conductive elements (electrodes, active mass, connectors and tabs).

### 2.1. Ohm’s Law

The internal resistance can be calculated from the current-voltage relationship given by two points in a discharge curve, like explained for lead acid batteries in [11,12]:

(2) $$R_{i}=\frac{U_{1}-U_{2}}{I_{2}-I_{1}}$$

The change of the voltage is observed during a cell operation in three possible states as defined by Kobayashi *et al.* [17]:

- during discharge of cell
- during recharge of cell
- during the interruption of current flow (charge/discharge)

The influence of different discharge times during determination of the internal resistance by current switching methods is already explained for lead acid batteries [13]. It was determined that the measurement outcome is strongly influenced by the discharge time, discharge current and state of charge of the battery [13]. However, the direct current method is only suitable for pure ohmic elements during the discharge or recharge of the battery and is not valid for complex elements like inductors or capacitors, according to Ratnakumar *et al.* [3].

### 2.2. Joule’s Law

Current flowing through a battery generates heat. For all batteries the value of heat is defined by the I^2^R_i_ losses resulting from current flows through the internal resistance of the battery during the charging and discharging processes [14–16]. This heat development effect caused by the internal resistance is also known as Joule heating. From this effect it is possible to calculate the internal resistance by determining the heat loss during the cell operation. A calorimeter, for example, can be used as a device for measurement of heat from chemical reactions or physical changes [4,15,17] even for high power cells with low heat generation:

(3) $$Q_{\mathrm{loss}}=I^{2}\cdot R_{i}\cdot t$$

In addition to Joule’s heating, the chemical reactions which take place in the cells may be exothermic, adding to the generated heat, or they may be endothermic, absorbing heat during the process of the chemical reaction [15,17]. For lithium ion batteries the reversible heat effect during charging, caused by the reaction (intercalation of Lithium ions to the lattice) is initially endothermic, then turns to slightly exothermic during most of the charging cycle. During discharge, the reaction is reverse. Under high current load conditions, the Joule heating effect at lithium ion cells is greater than the reversible heat effect, therefore during the determination of internal resistance by calorimeter measurements, those effects were not taken into account [2]. If a battery is cycled symmetrically (equal amounts of charge and discharge) around a given SOC, the reversible heat effect is cancelled out, and only Joule’s law governs heat dissipated by the battery. If this is the case, internal reactance of the battery can be determined by Joule’s heating with a calorimeter.

**Figure 1.**
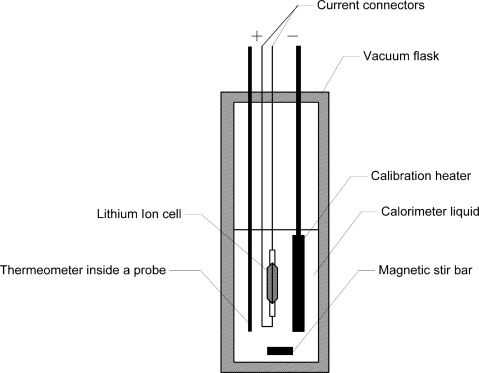
Quasi-adiabatic calorimeter used for determination of battery heat generation.

Figure 1 shows the basic elements of a typical calorimeter, as introduced by Uchidab *et al.* [17]. Its wall is an ideal thermal insulator (Dewar vessel). The system (equipment) under test (EUT) is in our case a single lithium ion cell. Its internal resistance acts as an electrical heater R, which creates the heat during charge and discharge process. The complete device comprises of a stirrer and a thermometer plunged in a liquid that surrounds the system to be investigated.

Heat dissipated by the cell can be calculated by the temperature increase, assuming that the heat capacity of the whole setup is known. The heat capacity of the setup can be determined by injection of a known amount of heat into the system by the calibration heater.

C_cal_ is the heat capacity of the calorimeter. For the calculation of the value of R_i_ from the known values of C_cell_, C_cal_ ΔT, Q one needs to determine the temperature change during the charge/discharge cycle of the cell by calorimeter measurements with a substance (“calorimeter liquid”) and while using a lithium ion cell whose heat capacities have been measured by previous tests.

Due to the characteristics of the cell (limited energy) under test, Equation (3) and the determinate Equation (4) for this calorimeter test with a constant current are not applicable for long term measurements. Therefore an alternating current was used for creating heat dissipation, as shown in Equations (5) and (6):

(4) $$Q=I^{2}\times R\times t=\left(C_{\mathrm{cal}}+C_{\mathrm{cell}}\right)\times \Delta T$$

(5) $$Q_{\mathrm{loss}}=\int_{t_{1}}^{t_{2}}{\left(I\left(t\right)\right)}^{2}\cdot R_{i}\cdot dt$$

(6) $$Q_{\mathrm{loss}}=\int_{t_{1}}^{t_{2}}{\left(I\left(t\right)\right)}^{2}\cdot R_{i}\cdot \mathrm{dt}=\left(C_{\mathrm{cal}}+C_{\mathrm{cell}}\right)\times \left(T_{2}-T_{1}\right)$$

Internal resistance of the cell can be calculated by transforming Equation (7):

(7) $$R_{i}=\frac{\left(C_{\mathrm{cal}}+C_{\mathrm{cell}}\right)\times \left(T_{2}-T_{1}\right)}{\int_{t_{1}}^{t_{2}}{\left(I\left(t\right)\right)}^{2}\mathrm{dt}}$$

### 2.3. AC Resistance

The AC resistance of a battery is measured by providing the battery with a small alternating current (AC) I(t) ripple that generates a small AC voltage U(t) response e.g. with a constant frequency of 1 kHz. Electrical impedance is the relative ratio of the variation in current with the variation in voltage, as showed in Section 2.3.4 “Dynamic modelling of batteries” [18]. The impedance describes not only the relative amplitudes of the voltage and current (like at the DC internal resistance), but also the relative phases. In comparison to the direct current (DC) method (switch in/switch off) the AC method is additionally defined by the phase angle [3,18]:

(8) $$Z=\frac{U(t)}{I(t)}=\frac{U_{\text{max}}e^{i(\omega t+\varphi_{U})}}{I_{\text{max}}e^{i(\omega t+\varphi_{I})}}$$

The measured electrical impedance (complex resistance) is generally dependent on the frequency of AC current used for the measurements. Different devices used for the determination of the electrical impedance may create a significant variance of the results. The AC current method is suitable for complex resistances.

### 2.4. Electrochemical Impedance Spectroscopy (EIS)

Like in the simple AC impendence determination, during electrochemical impedance spectroscopy (EIS) a small amplitude AC signal is also applied to the cell. EIS can provide detailed information of the cell under examination; parameters such as corrosion rate, electrochemical mechanisms and reaction kinetics, detection of localized corrosion, battery life and of course internal resistance (impedance) [8,18].

**Figure 2.**
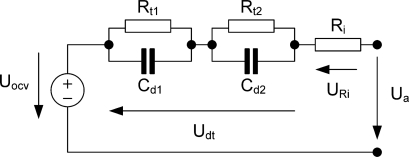
Equivalent circuit model of a battery [16].

The equivalent circuit (Figure 2) shows an ohmic resistance (R_i_), which is a function of the battery contacts impedance, the inter- and intra-cell connections, the electrodes and the electrolyte, the SOC (state of charge) of the battery, the battery ageing history and the battery temperature. R_t1_ and C_d1_ are the charge-transfer resistance and the interfacial capacitance (which includes the double layer capacitance and associated capacitive components due to adsorption, passive films, *etc.*) for the first electrode, and R_t2_ and C_d2_ are the charge transfer resistance and interfacial capacitance for the second electrode [19].

## 3. Experimental Section

An ENAX (ENAX Inc., Tokyo, Japan) “High Power Cell” type NX2P0M lithium ion cell (2.0 Ah nominal capacity at 1C-rate) has been used in this study. This type of cell is designed for high power and lifetime demands, and also integrates a ceramic separator for high safety design. The negative electrode consists of a special carbon modification in order to meet the high power demands, especially during charging mode. The positive electrode is manganese-based for safety, power and cost issues. The cell shows a very robust behavior making it to an excellent candidate for the present study. At a current rate of 20 C the cell showed a capacity of 1.8 Ah, which has been used in the following as reference capacity for the cell.

DC internal resistance measurements and battery cycling for energy loss and calorimetry were done with a BNT 300-05-4 battery test system from Digatron Firing Circuits (Aachen, Germany). The cell was connected by a four wire connection to the Digatron system. The wire gauge of the copper cables was 95 mm^2^. The accuracy of the measurement is 0.05% (end of scale) value in the range of 1% to 10% maximum value (I = 3 A to 30 A; U = 0.05 V to 0.5 V) and 0.5% of reading in the region of 10% to 100% of maximum value (I = 3 A to 30 A; U = 0.05 V to 0.5 V). The data acquisition with the Digatron was carried out for all parameters (voltage, current, temperature) with a sample rate of 100 Hz. Curve shape and rise time of the current pulses were analyzed with a Tektronix TDS3014B (Tektronix, Beaverton, OR, US) oscilloscope. Slowest observed rise time was 20 ms.

All measurements were carried out at 25 °C. Temperature was controlled by a temperature test chamber (Vötsch VT 4004, Vötsch Industrietechnik, Balingen-Frommern, Germany) which was used to maintain the desired cell temperature within ±0.5K. The cell was placed between two aluminum cooling profiles (R_th_ = 0.6 K/W, Type 938SP-01500-A-200 HS Marston, Wolverhampton, UK), to keep its temperature at 25 °C. Temperature deviation caused by current profiles was within deviations caused by the temperature chamber. Only during calorimetric measurements were these profiles not used. To ensure comparable experimental results, all measurements were carried out at the same charge condition of 60% SOC. To ensure this condition, the cell was discharged and charged with standard rate of 1 C until 100% SOC, followed by a 1 C discharge rate to the designated SOC of 60% and a 30 minutes rest period to ensure that the cell has reached the desired temperature.

Fixed frequency AC impedance was measured with a Hioki 3554 resistance meter (Hioki, Nagano, Japan). The current amplitude for this measurement was 150 mA. Model 9465-10 PIN TYPE LEAD connectors were used for connecting the cell. Typical accuracy of this instrument is 1.0% rdg.

AC impedance spectra were measured with an IM6ex Workstation (Zahner Electric, Kronach, Germany). The measurement was carried out in galvanostatic mode with a 1 A amplitude and in a frequency range from 50 mHz to 200 kHz at lower limit with 4 steps per decade and above 66 Hz with 10 steps per decade. Cell was connected by a four wire connection with the LoZ Cable Set (Zahner).

Calorimetric measurements were carried out with an in-house made quasi-adiabatic calorimeter instead of the isothermal calorimeter described in literature as typically used for battery measurements [4]. The battery calorimeter consists of a double-walled Dewar-Vessel with a volume of 10 dm^3^ (Nalgene type 4150, Thermo Fisher Scientific, Waltham, MA, US). This vessel provides an excellent thermal insulation towards the surrounding. The calorimeter was filled with approx. 5 dm^3^ of deionised water. This type of calorimeter is called quasi-adiabatic because heat transfer is strongly reduced by a good insulation to the surrounding but not completely eliminated. Heat transfer is zero for a true adiabatic calorimeter. This is ensured by heaters inside the wall of an adiabatic calorimeter. The water in the vessel is mixed by a magnetic bar (Heidolph Komet from Heidolph, Kehlheim, Germany) and a magnetic stirrer (Heidolph Hei-Mix D) to ensure thermal equilibrium in the water. The temperature was measured with a thermistor based thermometer. Instead of the thermometer described in reference [20] a Betatherm 100K6A1B thermistor (Measurement Specialties, Hampton, US) and a 1 MΩ resistor type RC55D1M0BB (Welwyn, Welwyn, UK) with an accuracy of ±0.1% has been used. Temperature can be measured in the range from 0 °C to 70 °C with a resolution of ±30 mK. The calorimeter is equipped with a calibration heater. The heat capacity of the experimental setup, including the battery and water, was measured with this heater by heating the setup with a defined amount of electric energy. The heater consists of spiral wound Konstantan wire (2.5 mm thickness, 2.2 m length, 220 mΩ resistance). To generate the heat for calibration, a constant current of 15 A was supplied by the Digatron system to the heater. Voltage and current of the heater were recorded by Digatron. The heat introduced into the calorimeter was calculated by integration of voltage and current with time. During the heating period, the temperature inside the vessel was measured. The heat capacity of the setup can be easily calculated by the temperature increase and electric energy generated from the heater. Accuracy of the energy measurements by the calorimeter of about 5% was determined by error calculation.

## 4. Results and Discussion

Internal resistance of the lithium ion cell was measured by the following methods under the conditions described above:

- VDA current step method
- ISO current step method
- Similar to DIN EN 62391-1
- Current-off method
- Switching current method
- AC internal resistance
- Impedance spectroscopy
- Energy loss method
- Quasi-adiabatic calorimeter

The results of the measurements are discussed in detail in the following sections.

### 4.1. VDA Current Step Method

**Figure 3.**
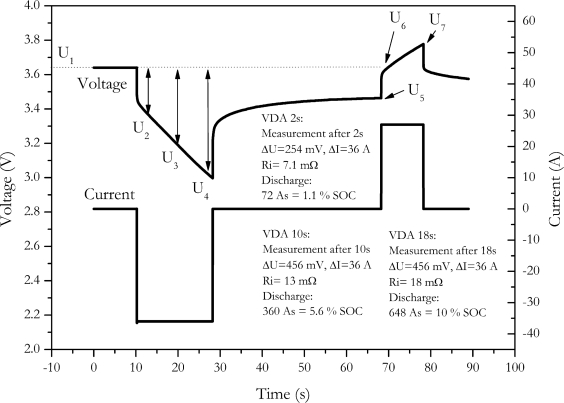
Measurement of internal resistance according VDA test procedure.

The VDA (Verband der Automobilindustrie, Frankfurt am Main, Germany) provides a collection of measurement procedures for cells and batteries for automotive applications [21]. According to the VDA procedure, the discharge resistance of the cell is measured by a constant current discharge pulse with duration of 18 seconds and a current rate of 20 C. After rest period of 40 seconds, a 10 seconds charge pulse of 16.6 C is applied to the cell to measure charge resistance. This current profile was applied to the cell NX2P0M and the resulting cell voltages are shown in Figure 3. Discharge resistance is calculated by the resulting voltage drop after 2 seconds, 10 seconds and 18 seconds after current is switched on [21]:

(9) $${\mathrm{Ri}}_{\mathrm{discharge},2s}=\left|\frac{U_{1}-U_{2}}{I_{\mathrm{discharge}}}\right|$$

(10) $${\mathrm{Ri}}_{\mathrm{discharge},10s}=\left|\frac{U_{1}-U_{3}}{I_{\mathrm{discharge}}}\right|$$

(11) $${Ri}_{\mathrm{discharge},18s}=\left|\frac{U_{1}-U_{4}}{I_{\mathrm{discharge}}}\right|$$

Charge resistance is calculated the same way, but the voltage drop is referenced to the voltage just before the charge pulse [21]:

(12) $${\mathrm{Ri}}_{\mathrm{charge},2s}=\frac{U_{6}-U_{5}}{I_{\mathrm{charge}}}$$

(13) $${\mathrm{Ri}}_{\mathrm{charge},10s}=\frac{U_{7}-U_{5}}{I_{\mathrm{charge}}}$$

The measurement results are shown in Table 1. To generate a significant voltage drop, the VDA procedure applies a rather high current to cell. Due to this high current, significant amount of charge is either withdrawn from the cell or charged back into the cell by the measurement pulse.

A substantial increase of the calculated resistance is observed with increase of the pulse length. This is observed at the discharge pulse as well as at the charge pulse. This feigned increase of internal resistance is mainly caused by the voltage change due to the discharge or charge of the measuring pulse.

In Table 1 the amount of change of the SOC of the battery caused by the pulse is shown. Up to 10% of the battery’s capacity is discharged during the discharge pulse. Since pulse duration and current of the charge pulse are lower, only up to 4.6% of the battery’s capacity is charged during the charge pulse. However, by changes in that high amount of current a significant change of the voltage results. This is not only caused by internal resistance effects but also by changing the state of charge, therefore the resistance values calculated by these pulses are strongly falsified because of the battery’s changing state of charge. Though this effect is described in detail by Ratnakumar *et al.* [3], no hints for solving this problem were given.

Thus, to obtain meaningful values for internal resistance, the charge withdrawn from the battery should be reduced. Therefore the additional voltage drop introduced by SOC should be effectively eliminated. In principle, the voltage drop introduced by SOC change can be corrected if the OCV after the pulse is used for determining the voltage change introduced by the SOC change. To get a stable OCV a rest period of at least 15 minutes must be kept. To perform the experiment in a reasonable time, this can be achieved by two ways.

In the first way the current can be reduced, but if the amplitude of the pulse is reduced, the voltage response is also reduced. Since the internal resistance is calculated by the difference of two voltages, a strong increase of measurement uncertainty will result.

**Figure 4.**
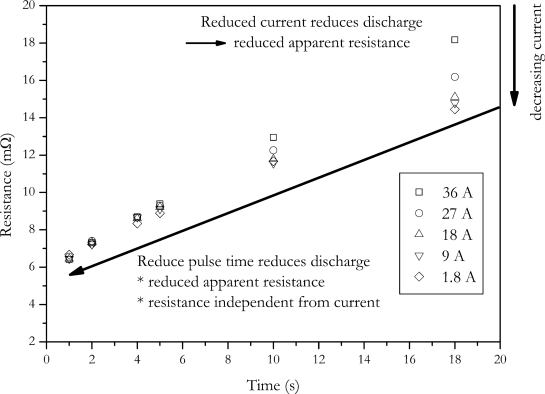
Dependence of calculated internal resistance on pulse duration and pulse amplitude.

In the other way, the amount of charge change is reduced by reducing the pulse duration. In Figure 4 the dependence of calculated internal resistance on pulse duration and pulse amplitude is shown. The data of this Figure was generated by applying the VDA pulse to the cell with different current amplitudes. Internal resistance was calculated according to Equation (2). The voltage U_2_ was measured at times shown in Figure 4.

The data shows that the calculated internal resistance is increased with current and pulse duration. This is due to the fact that the feigned resistance generated by the voltage drop caused by the change of SOC is reduced with reduced pulse amplitude and pulse duration. The present data also shows that at short pulse durations the influence of current disappears. This is also caused by the same effect, but here no feigned resistance is introduced by changing battery’s charge [3].

As stated above, the accuracy of the measurement is related to the measurement current. The impact of the amplitude of the measurement current to the accuracy can be calculated by the error propagation law. The error of the measurement is determined by the error of current measurement, ΔI, the error of the voltage measurement, ΔV, as well as the error of the temperature control due to the temperature dependency of the internal resistance. Temperature increase of the cell caused by current pulses was neglected for the error calculation, because measurement cell was placed between two aluminium cooling profiles with low thermal resistance. With this setup temperature increase was below measurement accuracy.

The measurement deviation of internal resistance can be calculated by the following equation:

(14) $$\Delta R=\left|2\frac{U_{1}-U_{2}}{I^{2}}\right|\left|\Delta I\right|+\left|\frac{1}{I}\right|\left|\Delta U_{1}\right|+\left|\frac{1}{I}\right|\left|\Delta U_{2}\right|+\left|T_{c}\right|\left|\Delta T\right|$$

**Figure 5.**
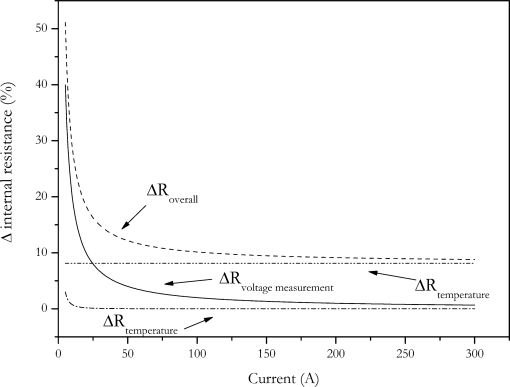
Accuracy of internal resistance measurement in dependence of pulse amplitude calculated by error propagation law. Contributions of uncertainties of current and voltage measurement and the thermal equilibration of the battery are also shown.

The error dependence of internal resistance with current is shown in Figure 5. For this calculation an internal resistance of 8 mΩ has been assumed.

As expected, the error of the internal resistance strongly increases with decreasing pulse current. Because of the strong increase in measurement error, reducing pulse current is not a solution for the charge change problem of the internal resistance measurement.

### 4.2. Optimized VDA Step Method

At first glance, the measurement accuracy seems to be independent from the pulse duration. Because of the limited bandwidth of the power stage of the measurement equipment, pulse duration cannot be infinitely reduced. The typical rise times of power stages of battery test systems are in the range from 1 ms to 10 ms. To ensure accurate measurement results, pulse duration should be well above this range.

If the voltage drop is measured 100 ms after the start of the current pulse, the change of charge of the battery is in the range from 0.05 to 0.06% and can therefore be neglected. For the NX2P0M cell an internal resistance of (4.5 ± 0.7) mΩ is found with the discharge pulse and (5.0 ± 0.8) mΩ is found with the charge pulse, (Table 1). Therefore, 100 ms should be enough settling time, even for slow test equipment.

### 4.3. Reducing Time, Advanced Method

**Figure 6.**
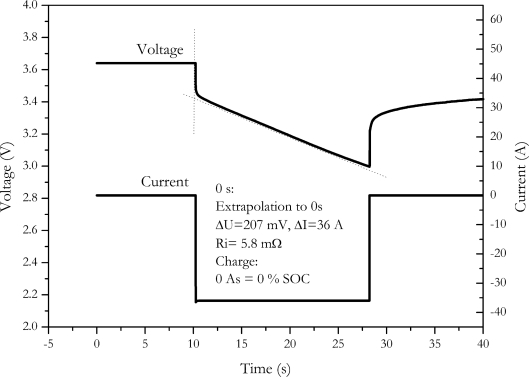
Reduction of feigned resistance introduced by discharge by extrapolation to the beginning of the discharge pulse.

The choice of 100 ms measurement time after voltage drop may nevertheless be a bit arbitrary. It was additionally found that the slope of the voltage during the pulse is nearly linear, as shown in Figure 6.

Therefore, the internal resistance can be calculated by extrapolating the voltage slope to the beginning of the discharge pulse. A least square fit of a linear curve is used for this extrapolation. This procedure is similar to the procedure described by the DIN EN standard for double layer capacitors [22]. By this well defined method, influences of discharge or charge, as well as the influence of the slew rate of the instrument are effectively cancelled out. With this method, we found a resistance of (5.8 ± 0.9) mΩ for the NX2P0M cell.

### 4.4. Current-Off Method

**Figure 7.**
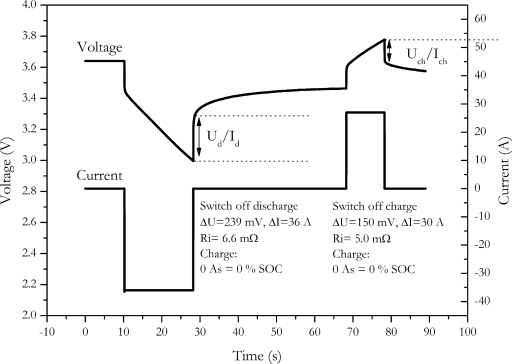
Measurement of internal resistance by switching off the pulse current.

Another way to eliminate the influence of charge change is the current-off method. Here the internal resistance is calculated by the voltage change resulting from the switch off of the current. An example of the method is shown in Figure 7.

Since the SOC is determined at the end of the current pulse, the internal resistance is not measured at the desired SOC of the battery. This might be a major drawback if the internal resistance of the battery is strongly SOC dependent. The choice of the second point for calculating the voltage difference is, however, also quite arbitrary, because the voltage curve of the battery is not well defined.

This measurement procedure can be applied for discharge as well as for charge pulses. For NX2P0M cell we found an internal resistance of (6.6 ± 1) mΩ with the discharge switch off and (5.0 ± 0.8) mΩ with the charge switch off.

### 4.5. Current Switch Method

**Figure 8.**
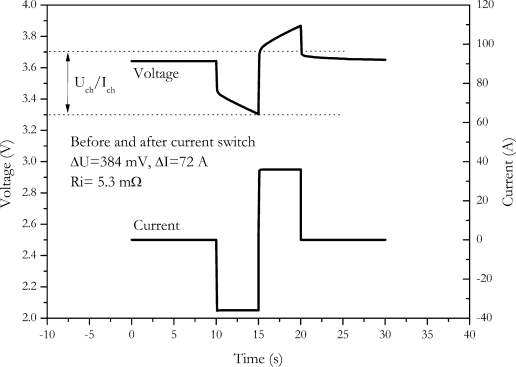
Measurement of internal resistance by switching current from discharge to charge.

Another method without influence on the charge to the outcome of measurement is the current switch method. A discharge is directly switched to charge. An example of this method is shown in Figure 8.

The main benefit is the increased voltage caused by the switch. Since a direct switch from discharge to charge occurs, the current amplitude is doubled. The voltage response is also doubled. A reduced error results due to the increased voltage response. Similar to the current-off method, the SOC is not measured at the starting SOC. Since a change in the sign of current occurs, this method is very demanding to the slew rate of the power stage of the measurement equipment. For the NX2P0M cell we found an internal resistance of (5.3 ± 0.5) mΩ with this procedure.

### 4.6. Energy Loss

If no side reactions or other heat effects occur in the cell, any heat generated in the cell is caused by the Joule effect [15,23]. This effect is directly linked to the internal cell resistance [15,23]. An ideal lithium cell nearly shows no side reactions within the specified load and temperature range. Under high current conditions, the reversible heat effect can by neglected for lithium ion cells, because it is far less than the heat generated by power dissipation from internal resistance. By measuring the charge and discharge energy at a charge neutral profile, the energy efficiency and the energy loss due to internal resistance can be calculated.

We chose a constant current charge/discharge profile for measuring the internal resistance with the energy loss method. Current amplitude was 36 A, pulse duration 5 seconds. This profile has been applied for 40 minutes to the cell. Charge and discharge energy was calculated by the software of the Digatron tester.

The loss of energy Q_loss_ is the difference between the charge and discharge energy. For the NX2P0M cell we found a loss of energy of 16.3 kWs. Internal resistance can be derived from Equation (6):

(15) $$R_{i}=\frac{Q_{\mathrm{loss}}}{\int_{t_{1}}^{t_{2}}{\left(I\left(t\right)\right)}^{2}\mathrm{dt}}$$

Resulting in an internal resistance of (5.3 ± 0.8) mΩ for the cell with this method. Within measurement accuracy this is the same value as found with the current step methods where no change of charge is involved.

### 4.7. Quasi-Adiabatic Battery Calorimeter

**Figure 9.**
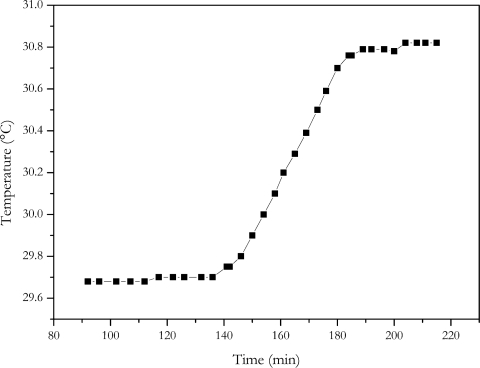
Temperature increase of the inside the calorimeter.

A calorimeter provides direct measurement of the heat generated by the current flowing through a cell. The same current profile as with the energy loss method was applied to the cell. The temperature increase of the calorimeters inside caused by the current profile is shown in Figure 9.

With a heat capacity of 15.8 kJ/K, a dissipated energy of 17.4 kWs was determined. According to Equation (1) an internal resistance of (6.5 ± 0.5) mΩ results for the NX2P0M cell. Within the range of measurement error, the calorimeter measurement gave the same result as the electric energy loss method. This is also the same value, within measurement accuracy, as determined with the current step methods where no change of charge was involved.

### 4.8. AC Internal Resistance

Measurement of internal resistance at fixed frequency with AC damping is common in practical applications. It is a convenient method involving low power and handy instruments. On the benefit side it is a fast method with no deterioration of the cell. Typically these measurements are carried out at 1 kHz. We found an internal resistance of (2.3 ± 0.3) mΩ for the NX2P0M cell. Since a low damping current is usually used for this type of measurement (typically in the range mA), only a low voltage response of the cell is generated. This limits the accuracy of the measurement method.

Since the AC impedance of a battery is strongly dependent on frequency, the comparison of internal resistances of different batteries, measured at different frequencies, is useless. The value found with this method completely differs from the values found by step methods as well from the values found by the energy dissipation methods. This finding is assigned to the complex impedance of a lithium ion cell and to the complete different time domains of the AC internal resistance measurement and the step methods.

### 4.9. Impedance Spectroscopy

The frequency dependence of the impedance of a cell is investigated with impedance spectroscopy. With this method, a frequency range from 1 mHz to 200 kHz is typically covered. This method gives detailed information about the behaviour of the cell. Despite of huge information gathered by impedance spectroscopy, a big advantage of this method is that there is no distortion by the measurement, since only low power is utilized.

Impedance spectroscopy also requires quite expensive equipment, and the measurement is generally time consuming, therefore this method is not suitable for analyzing a huge number of cell, e.g., at 100% quality checks, especially if low frequencies are included.

**Figure 10.**
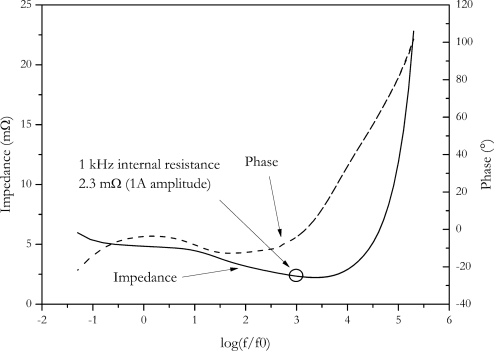
Bode plot of the NX2P0M cell, f0 = 1 Hz.

In Figure 10, the Bode plot of the NX2P0M cell is shown. At 1 kHz, the same internal resistance (2.3 ± 0.3) mΩ is found as with the direct AC resistance method, although internal resistance was measured with the Hioki resistance meter. This instrument also applies a small signal for measurement, so similar outcome of measurement is expected.

Accuracy of impedance spectroscopy is influenced by parasitic reactance, instrument set-up and principle uncertainty due to the limited frequency selectivity of the measuring method [24,25]. The limited frequency selectivity is usually the dominating effect.

**Figure 11.**
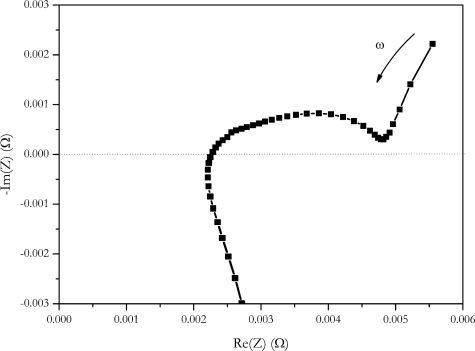
Nyquist plot of the NX2P0M cell.

Impedance found at low frequencies is in the range of 5 mΩ. It is in the same range as the current step methods. If impedance data is represented b a Nyquist plot (Figure 11), a value of 4.9 mΩ of the real part of impedance is found at the intersection of the semicircle and the Warburg straight line.

According to references [26,27] this is assigned to the sum of resistances of the electrolyte, the solid electrolyte interface and electron transfer reaction. Uchida *et al*. correlated this intersection with the heat generated by the cell [2]. Despite this fact, a similar value was found for internal resistance at these frequencies, and we believe, comparing the values found here with the step methods must be done with care because step methods are not low frequency methods. Current is switched at high slew rates with these methods. High slew rates in time domains correspond to high frequencies in the frequency domains. The slew rate of the Digatron system used for the current step is in the range from 10% to 90%, I_max_ is 5 ms, the slew rate from charge to discharge is 10 ms. This rate corresponds to a frequency of 200 Hz respectively 100 Hz. According to Fourier, rectangular steps contain frequencies up to an infinity. Because of the rectangular shape of the current pulse, no assignment to a discrete frequency can be made.

Table 2 summarizes the comparison of the results of all different methods.

Step methods without change of charge, energy loss methods and intersection of the impedance spectrum, give internal resistances in the range from 5 to 6 mΩ, so these methods are suitable for the prediction of the losses and the power ability of a lithium ultra high power cells. Because the polarization caused by diffusion through SEI and charge transfer are fast at these type of cells no separation between the ohmic resistance caused by electrolyte, electric conductivity of the active material and the conductors an the polarisation causes by diffusion through SEI and charge transfer can by made.

## 5. Conclusions

Several methods for the determination of internal resistance of lithium ion batteries were used to measure the internal resistance. It was found that a feigned resistance is occurring by charging or discharging the battery when the internal resistance is determined by the voltage drop of long and high current charge or discharge pulses. Reduction of pulse current and pulse duration was determined to reduce the influence of discharge and charge on creating a feigned resistance. An error estimation showed that reducing the pulse amplitude leads to unacceptable large measurement uncertainties. However, these uncertainties can be kept in an acceptable range by using high current pulses and using the voltage just after the start of the current pulse used for calculating the internal resistance. On one hand, a delay of 100 ms between start of the current pulse and voltage measurement enables a stable current output from the power stage of typical battery testing systems. On the other hand, 100 ms is short enough to prevent unintended change of charge of the battery, so the feigned resistance is negligible. Extrapolation to zero delay may be used to improve measurement accuracy but within the accuracy of the equipment used in this study an improvement cannot be stated clearly.

It has been shown that methods using steps give the same results, within measurement accuracy, if the outcome of measurement is not falsified by charging or discharging the cell. The outcome of measurement is independent of the sign of current. It is also independent if the voltage difference at the beginning or the end of the current pulse is used to calculate internal resistance of the cell. A direct switch, from charging to discharging, gives the same result as the other step methods.

The internal resistance of the cell was also determined by measurement of the waste energy caused by high current cycling. Waste energy was measured by the difference of charge and discharge energy as well as by determining the dissipated heat of the cell using a calorimeter. Both methods give the same results for internal resistance, within measurement range.

It was found that both energy loss methods give the same results for internal resistance as do current step methods (without change of charge). This is a clear hint that reversible heat effects of the cell are cancelled out if a symmetrical current profile is used. Because of the fact that two measurement principles give the same results for internal resistance, it is expected that the values determined with these methods provide reliable information about the pure ohmic resistance of a cell.

The method of measuring AC resistance at fixed frequency gives fast results. Because each cell type shows individual frequency dependence of impedance, AC resistance measured at different type of cells cannot be generally used for cell comparison and benchmarking. Therefore this method is only suitable for measuring and comparing internal resistance of the same type of cell, e.g., for quality screening.

However, at frequencies about 1 kHz AC-resistance for different cells (which have comparable size) has very similar frequency dependence. It also could be used for comparing different cells (relatively). It may be expected that cells with higher small signal resistance also have higher large signal resistance.

Despite the fact that the impedance spectrum of the cell was recorded with higher damping than the measurement at fixed frequency, the same result for 1 kHz was found. Comparison between values found with AC methods with the values found by step methods is not easy, because of the complex electrochemical nature of the cell and broad range of frequencies found in frequency domain.

Since the fast steps and the energy loss methods provide the same results, these methods thus recommend themselves for the measurement of internal resistance for designing the battery system, selecting and comparing cells, for energy efficiency calculation, for dimensioning the cooling system of the battery and for performing power estimation.

Determining the internal resistance by the voltage drop after a short delay after the start of a current pulse is a fast and convenient method for the measurement of internal resistance during storage tests, cell benchmarks and other highly automated cell screening purposes. Since values generated by these method are conform to the values from energy loss methods, they can replace the time consuming energy loss methods by a fast and easy method, speeding up the development process of energy storage devices for automotive applications.

## Figures and Tables

**Table 1.** Internal resistance of the NX2P0M cell, measured according to VDA procedure and the optimized procedure.

	**I/A**	**ΔU/V**	**R_i_/mΩ**	**ΔR_i_/mΩ**	**ΔQ/As**	**ΔSOC/%**
R_i,discharge, 2s_	36	0.254	7.1	1	72	1.1
R_i,discharge, 10s_	36	0.456	13	2	360	5.6
R_i,discharge, 18s_	36	0.648	18	3	648	10
R_i,charge, 2s_	30	0.203	6.7	1	60	0.9
R_i,charge, 10s_	30	0.346	12	2	300	4.6
R_i,discharge, 0.1s_	36	0.161	4.5	0.7	3.6	0.06
R_i,charge, 0.1s_	30	0.150	5.0	0.8	3.0	0.05

**Table 2.** Comparison of the outcome of internal resistance measurement with different methods on the same NX2P0M cell, all measurements were carried out at 60% SOC and 25 °C.

**Method**	**Execution**	**Ri/mΩ**	**ΔRi/mΩ**
Steps with change in cells charge	2 seconds, discharge	7.1	1
	10 seconds, discharge	13	2
	18 seconds, discharge	18	3
	2 seconds, discharge	6.7	1
	10 seconds, discharge	12	2

Steps without change in cells charge	100 ms discharge	4.5	0.7
	Discharge, extrapolated	5.8	0.9
	100 ms charge	5	0.8
	discharge off	6.6	1
	charge off	5	0.8
	current switch	5.3	0.5

Energy loss	Watt hour counting	5.3	0.8
	Calorimetry	6.5	1

AC methods	AC @ 1 kHz	2.3	0.3
	Impedance spectrum @ 1 kHz	2.3	0.3
	Impedance spectrum, intersection	4.9	0.3
